# The complete chloroplast genome of *Pulsatilla campanella* Fischer ex Krylov. (Ranunculaceae, *Pulsatilla* Miller)

**DOI:** 10.1080/23802359.2022.2087556

**Published:** 2022-06-23

**Authors:** Hefei Xue, Yueyue Song, Yanyun Yang, Che Bian, Liang Xu, Tingguo Kang

**Affiliations:** a School of Pharmacy, Liaoning University of Traditional Chinese Medicine, Dalian, China; b Key Laboratory of Traditional Chinese Medicine Research and Development of Hebei Province, Institute of Traditional Chinese Medicine, Chengde Medical University, Chengde, China

**Keywords:** *Pulsatilla campanella*, chloroplast genome, phylogenetic, Ranunculaceae

## Abstract

The complete chloroplast genome of *Pulsatilla campanella* Fischer ex Krylov was sequenced and reported for the first time. The length of the entire circular genome was 162,322 bp, and the GC content was 37.4%. There were 133 genes annotated, including 89 known protein-coding genes, 36 tRNAs, and 8 rRNAs. The complete chloroplast genome of *P.campanella* has consisted of two inverted repeat regions (IRs), a large single-copy region (LSC 82,087 bp), and a small single-copy region (SSC 17,497 bp). The phylogenetic tree was built based on 29 species, using the maximum-likelihood method. The results showed that *P.campanella was* clustered on the same branch with a variety of *Pulsatilla* plants. The data reveal the genetic relationship between the selected species and provide information for subsequent plant classification.

*Pulsatilla campanella* Fischer ex Krylov (FI. Ajan. 30. 1859) is an early spring plant with purple perianths, and its flower nodding before and at anthesis resembles a bell. It is mainly distributed across western Xinjiang, China, in addition to northern Mongolia and Russia's Central Asia. *P. campanella* is an important species in the genus *Pulsatilla*. Many species of the genus *Pulsatilla* have been used as a traditional medicine named ‘Bai Tou Weng,’ which has a long history and significant therapeutic effect in clinical practice (Kumar et al. 2008). The roots of *P. campanella* are used to treat bacillary dysentery and lymphatic tuberculosis in local Chinese herbal medicine records (Jiangsu New Medical College 1977; Li et al. 2019). Triterpene saponins proved to be the main active substances in *P. campanella* (Li et al. 1990). At present, there are many studies on chemical composition and pharmacological activities (Xu et al. 2012; Zhang et al. 2019), and the chloroplast DNA fragments for species identification have also been reported (Li et al. 2019). However, it is the first time to report on the sequencing of the complete chloroplast genome of *P. campanella*.

The experimental materials were collected from Tekes, Ili Kazak Autonomous Prefecture, Xinjiang, China (E 81°89′30″, N 43°26′98″), and were identified as *P. campanella* by professor Tingguo Kang (Liaoning University of Traditional Chinese Medicine, Dalian, China). We used 200 mg of fresh leaves to extract total genomic DNA using the modified CTAB method (Doyle and Doyle 1987). The total genomic DNA was constructed in a sequencing library with a 350 bp insert using the NexteraXT DNA library preparation kit (Illumina, San Diego, CA, USA), and double-terminal sequencing was performed on the library using the Illumina Novaseq 6000 sequencing platform. The raw data was edited using NGS QC Tool Kit v2.3.3 (Patel and Jain 2012). High-quality reads were assembled into chloroplast genome using a *de novo* assembler SPAdes v3.11.0. (Bankevich et al. 2012). Finally, it was annotated by PGA (Qu et al. 2019) with *Pulsatilla dahurica* (MK860685) as reference genome. All experimental contents were approved by the School of Pharmacy, Liaoning University of Traditional Chinese Medicine, Dalian, China (20200112). All operations were under guidelines from the Specification on Good Agriculture and Collection Practices for Medicinal Plants (GACP; Number: T/CCCMHPIE 2.1-2018). The voucher specimen and genomic DNA were kept in the herbarium of Liaoning University of Traditional Chinese Medicine (Xinlei Zhao and Liang Xu, 861364054@qq.com, *P.campanella* number: 10162200525972LY). The sequencing data were assembled using ABySS v2.0.2 (http://www.bcgsc.ca/platform/bioinfo/software/abyss). The protein-coding sequences of chloroplast were compared with the known protein databases (eggNOG, GO, KEGG, NR, Swiss-Prot) to predict protein-coding genes.

The entire genome is 162,322 bp in length. It has a typical circular structure, with a GC content of 37.4%. The genome contains 133 genes, including 89 known protein-coding genes, 36 tRNA genes, and 8 rRNA genes. The total length of the coding gene is 92704 bp, accounting for 57.11% of that total length of the genome. Besides, 17 genes (*rnK-UUU*, *rps16*, *trnG-UCC*, *atpF*, *rpoC1*, *trnL-UAA*, *trnV-UAC*, *petB*, *petD*, *rpl16*, *rpl2*, *ndhB*, *trnI-GAU*, *TrnA-UGC*, *ndhA*, *clpP*, *ycf3*) containing 19 introns has been found.

The phylogenetic tree is a commonly used method to visualize the evolutionary relationship between species. The complete chloroplast genomes of 29 species (including *P. campanella*) were selected to build a phylogenetic tree, using the maximum likelihood method and the model TVM + F+R4 in IQ-TREE 1.6.12 (Nguyen et al. 2015) with 1000 bootstrap replicates(Figure 1). In the process, *Panax Ginseng* existed as an outgroup. The phylogenetic tree showed that *Panax Ginseng* was distant from other species, and *P. campanella* was clustered on the same branch with a variety of *pulsatilla* plants, which confirms the authenticity and reliability of the results. The data will not only provide a basis for analyzing the phylogenetic position of *P. campanella*, but also lay a theoretical foundation for the systematic classification of Ranunculaceae.

**Figure 1.**
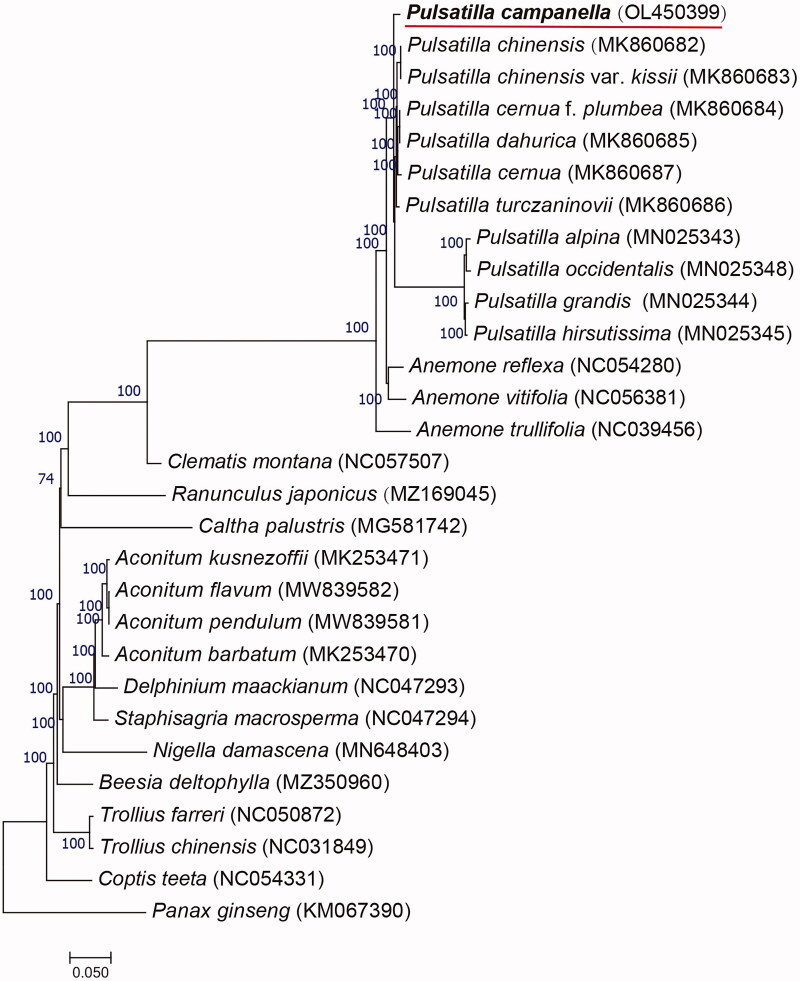
Maximum-likelihood (ML) phylogenetic tree of *P. campanella* and 28 other species. Numbers above the branches indicate the bootstrap values from ML analyses.

## Author contributions

Hefei Xue: Analysis of data, conception and drafting for the work. Yueyue Song: Revising critically for important intellectual content. Yanyun Yang and Che Bian: Acquisition and analysis of data. Liang Xu and Tingguo Kang: Final approval of the version to be published.

## Data Availability

The genome sequence data that support the findings of this study are openly available in GenBank of NCBI at (https://www.ncbi.nlm.nih.gov/) under the accession NO. OL450399. The associated BioProject, SRA, and Bio-Sample numbers are PRJNA778819, SRX13107158 (Illumina), and SAMN22999511, respectively.
